# Co-Occurrence and Combinatory Effects of *Alternaria* Mycotoxins and Other Xenobiotics of Food Origin: Current Scenario and Future Perspectives

**DOI:** 10.3390/toxins11110640

**Published:** 2019-11-03

**Authors:** Francesco Crudo, Elisabeth Varga, Georg Aichinger, Gianni Galaverna, Doris Marko, Chiara Dall’Asta, Luca Dellafiora

**Affiliations:** 1 Department of Food and Drug, University of Parma, Parco Area delle Scienze 27/A, 43124 Parma, Italy; francesco.crudo1@studenti.unipr.it (F.C.); gianni.galaverna@unipr.it (G.G.); doris.marko@univie.ac.at (D.M.); chiara.dallasta@unipr.it (C.D.); 2 Department of Food Chemistry and Toxicology, Faculty of Chemistry, University of Vienna, Währinger Str. 38, 1090 Vienna, Austria; elisabeth.varga@univie.ac.at (E.V.); georg.aichinger@univie.ac.at (G.A.)

**Keywords:** *Alternaria* mycotoxins, combinatory effects, food safety, combined toxicity, co-occurrence, bioactive compounds

## Abstract

Mycotoxins are low-molecular weight compounds produced by diverse genera of molds that may contaminate food and feed threatening the health of humans and animals. Recent findings underline the importance of studying the combined occurrence of multiple mycotoxins and the relevance of assessing the toxicity their simultaneous exposure may cause in living organisms. In this context, for the first time, this work has critically reviewed the most relevant data concerning the occurrence and toxicity of mycotoxins produced by *Alternaria* spp., which are among the most important emerging risks to be assessed in food safety, alone or in combination with other mycotoxins and bioactive food constituents. According to the literature covered, multiple *Alternaria* mycotoxins may often occur simultaneously in contaminated food, along with several other mycotoxins and food bioactives inherently present in the studied matrices. Although the toxicity of combinations naturally found in food has been rarely assessed experimentally, the data collected so far, clearly point out that chemical mixtures may differ in their toxicity compared to the effect of toxins tested individually. The data presented here may provide a solid foothold to better support the risk assessment of *Alternaria* mycotoxins highlighting the actual role of chemical mixtures on influencing their toxicity.

## 1. Introduction

Mycotoxins are low-molecular-weight toxic compounds synthetized by different types of molds belonging mainly to the genera *Aspergillus*, *Penicillium*, *Fusarium* and *Alternaria* [1]. They may enter the food chain worldwide as a consequence of the ability of mycotoxin-producing molds to infect a wide number of crops and food commodities [2]. It has been reported that up to 25 % of world crops may be contaminated with mycotoxins and over 4.5–5.0 billion people are thought to be chronically exposed to these food contaminants [3]. However, a much higher prevalence of detected mycotoxins can be found depending either on the considered mycotoxin or crop (up to 80% in certain circumstances), as recently reported [4]. Although the highest levels of food contamination are more frequently found in low-income countries, mycotoxins actually represent a growing threat also on account of climate changes [5]. The contamination of food and feed by mycotoxins results in significant economic losses worldwide, not only in terms of food and feed spoilage, but also in terms of a burden on human health, animal productivity and international trade [6]. In particular, mycotoxins may pose a toxicological concern for humans and animals since they may exert a wide number of effects including acute toxic, mutagenic, carcinogenic, teratogenic, estrogenic and immunotoxic actions [7]. Among the various categories of mycotoxins, those produced by the genus *Alternaria* are gaining increasing interest due to their frequent occurrence in food, the recent insights on their genotoxic potential and mechanisms of action, and their consequent possible effects on human health [2,7]. The *Alternaria* toxins belong to the group of the so called “emerging” mycotoxins. They are compounds of possible concern due to their abundance, occurrence or toxicity, but the limited available data do not allow a comprehensive risk assessment with an acceptable degree of certainty.

**Figure 1:**
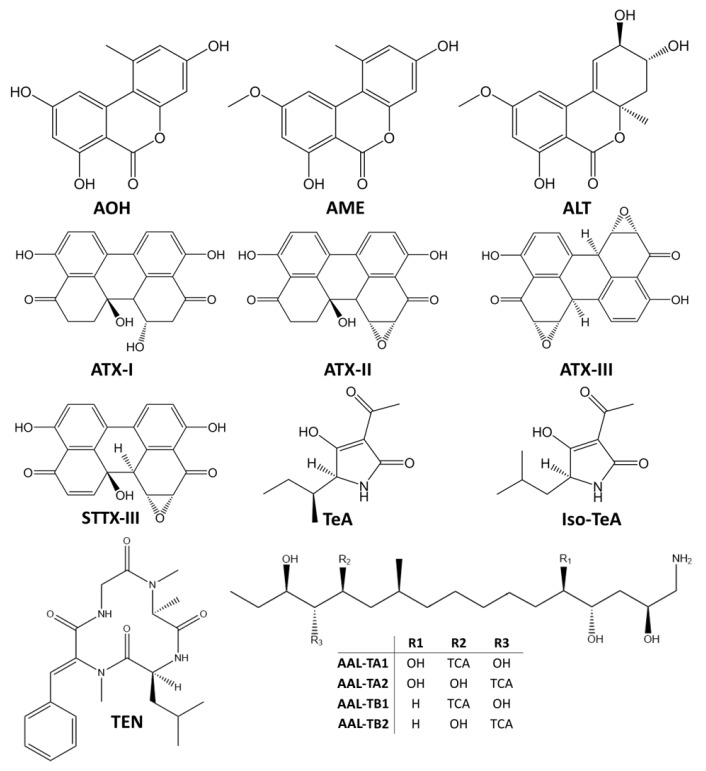
Chemical structures of the main *Alternaria* mycotoxins. AOH – alternariol; AME – alternariol monomethyl ether; ALT – altenuene; ATX-I, ATX-II, ATX-III – altertoxin I, II and III; STTX-III – stemphyltoxin III; TeA – tenuazonic acid; Iso-TeA – iso-tenuazonic acid; TEN – tentoxin; AAL-TA1-2 *Alternaria alternata* f. sp. *lycopersici* toxins sub-group A 1 and 2; AAL-TB1-2 *Alternaria alternata* f. sp. *lycopersici* toxins sub-group B 1 and 2; TCA - tricarballylic acid.

*Alternaria* species are ubiquitous plant pathogens and saprophytes that may contaminate a wide variety of crops and raw materials due to their environmental adaptability, particularly to their tolerance to low temperature and water stress conditions. They produce a cocktail of secondary metabolites and more than 70 *Alternaria* toxins have been characterized so far [2]. Based on their chemical structures, *Alternaria* toxins may be divided into five groups (Figure 1): (i) dibenzo-α-pyrones, including alternariol (AOH), alternariol monomethyl ether (AME), and altenuene (ALT); (ii) perylene quinones, including the altertoxins I, II, III (ATX-I, ATX-II and ATX-III, respectively), stemphyltoxin I and III (STTX-I and STTX-III, respectively), and alterperylenol/alteichin (ALP); (iii) tetramic acid derivatives, including tenuazonic acid (TeA) and iso-tenuazonic acid (iso-TeA); (iv) *A. alternata* f. sp. *lycopersici* toxins, which includes several phytotoxins such as AAL-TA and ALL-TB sub-groups (v) miscellaneous structures, as tentoxin (TEN), which has a cyclic tetrapeptidic structure [2,8]. However, many other mycotoxins might be produced by *Alternaria* spp. such as dihydrotentoxin, isotentoxin, altenuisol (ALTSOH), altenusin, infectopyrone, altersetin, macrosporin A, altersolanol A, monocerin, altenuic acids I, II, and III [9].

Due to the broad spectrum of adverse effects observed in vitro (e.g., genotoxic, mutagenic, clastogenic, androgenic, and estrogenic effects) and in vivo (e.g., fetotoxic and teratogenic effects), some of the *Alternaria* mycotoxins most frequently found in food may pose a severe threat to human health, especially for the most exposed categories such as infants, toddler and vegetarians [10]. Nevertheless, for most *Alternaria* mycotoxins, neither the toxicity nor the occurrence in food is adequately described. The current limitation of data hinders the proper assessment of risks to human health and, consequently, it prevents the establishment of specific regulations [11]. Therefore, the need of additional representative data to support the proper risk assessment of *Alternaria* toxins, especially for AOH, AME, TeA, TEN and ALT, was claimed by the expert Committee “Agricultural Contaminants” of the EU commission in 2012 [12]. In 2016, a call to collect data for the human exposure assessment to *Alternaria* toxins (AOH, AME, TeA and TEN) was published by the European Food Safety Authority (EFSA) [13].

In this respect, the chemical risk assessment of food-related compounds is currently based on the integration of knowledge about the single exposure to a given substance and its potential to individually cause harmful effects [14]. However, food is typically contaminated simultaneously by more than one mycotoxin. It is noteworthy that the simultaneous occurrence of compounds (either toxicants or bioactive food constituents) may lead to combinatory interactions (namely, additive, synergistic or antagonistic effects) that may significantly change the final toxicological outcome depending on the overall composition of chemical mixtures (see Section 3.2). In addition, mycotoxins may be present in food along with a high number of bioactive compounds, showing a huge variety of chemical structures and mechanisms of action, which may further modify their toxic impact. On this basis, risk assessment studies should take into account this complexity rather than relying on individual evidences, to better evaluate the overall risk associated with the consumption of mycotoxins-contaminated food.

Therefore, in the framework of supporting a better risk assessment of *Alternaria* mycotoxins, this work aims at consolidating the current knowledge on occurrence and combined actions of *Alternaria* mycotoxins. The relevance of investigating the effects and occurrence of chemical mixtures to support the thorough assessment of the actual risk this class of mycotoxins may pose to humans is pointed out. In more detail, this work presents the current state-of-the art in terms of co-occurrence and combinatory effects of: (i) different *Alternaria* toxins; (ii) *Alternaria* toxins in combination with other mycotoxins; (iii) *Alternaria* toxins in combination with bioactive compounds of food origin.

## 2. Natural Occurrence and Co-Occurrence of *Alternaria* Mycotoxins in Food

The occurrence of *Alternaria* mycotoxins in food and feed has been reviewed over the years [8,15,16,17,18]. However, in most cases, the occurrence and the relative concentrations of single or a small group of toxins has been reported, whilst the simultaneous co-occurrence of a high number of mycotoxins likely co-occurring together was not systematically assessed.

This section presents a collection of the co-occurrence of multiple *Alternaria* toxins in food commodities. In addition, data on the co-occurrence of *Alternaria* mycotoxins along with other mycotoxins and food constituents are reviewed. The key references covered in this review addressing the natural co-occurrence of different *Alternaria* mycotoxins are summarized in Table 1, while a schematic overview of the literature concerning the study of the co-occurrence of *Alternaria* and other mycotoxins is provided in Table 2. Detailed information concerning the number of samples analyzed, mycotoxin concentrations, as well as the methods and instruments used are reported in the Supplementary Materials (Table S1).

### 2.1. Co-Occurrence of Different Alternaria Toxins in Food

With regard to the co-contamination of food by different *Alternaria* toxins, AOH, AME, ALT, TeA, TEN, and ATX-I are the most frequently investigated compounds, while broader sets of compounds, including for instance ATX-II, IsoALT, AAL-TA1, AAL-TA2, ALP, macrosporin, ALTSOH, and Val-TeA, are rarely reported.

As shown in Table 1, the presence of *Alternaria* mycotoxins has been well-documented both in fresh and processed food, including fruits and vegetables, nuts, seeds, cereals, and fermented beverages. Among the food commodities investigated so far, apples, tomato, and their derivative products have been more frequently explored than other types of fruits and vegetables. Notably, most of them were found simultaneously contaminated by both AOH and AME, and, in some cases, also by up to five different mycotoxins. One of the first investigations were performed by Stinson and co-workers [19] who reported the contamination of apples and tomatoes with several *Alternaria* toxins already back in 1981. The observed contamination determined by HPLC-UV was in the low mg/kg range for AOH and AME, and in the µg/kg range for ALT and TeA in the case of apple samples. In tomatoes, TeA showed the highest contamination levels with up to 139 mg/kg. Furthermore, the presence or absence of ATX-I was assessed by thin layer chromatography. In the last ten years, multi-analyte measurements using liquid chromatography coupled to mass spectrometry became more and more important. The contamination with seven different *Alternaria* mycotoxins (AOH, AME, ALT, TeA, TEN, ATX-I, and ALP) and two phase-II metabolites (AOH-3-sulfate and AME-3-sulfate) was reported in tomato sauce, sunflower seed oil and wheat flour samples by Puntscher et al. [20]. In this study, the simultaneous contamination in the µg/kg range was reported in sample(s) from Austria, Croatia and Italy.

Infant foods were also found to be contaminated by multiple *Alternaria* mycotoxins. As an example, Gotthard and co-workers reported that tomato sauce and apple-pear-cherry puree were simultaneously contaminated by AOH, AME, TeA, and TEN [21]. In addition, those mycotoxins were also found in cereal-based infant formulas and they were reported along with ATX-I in wheat- and spelt-based food. These results are particularly relevant considering that the young population (infants and toddlers) show a higher exposure to *Alternaria* toxins in comparison to the other population categories due to their high food consumption in relation to body weight [10]. The most important dietary contributors to these mycotoxins were fruits and fruit products, vegetable oil, cereal-based foods and fruiting vegetables (tomatoes) wherein multiple mycotoxins were often found simultaneously, as shown in Table 1.

This scenario is further complicated by the possible presence of so called “masked mycotoxins”. This term refers to modified forms of mycotoxins as a result of their metabolic transformations in plants. Masked mycotoxins have been reported to abundantly co-occur in contaminated food and raw materials along with their respective parent counterparts [22]. The most common masked mycotoxins covalently link sulfate or glucoside groups as a result of plant phase-II metabolism [23]. After ingestion, these phase II plant metabolites can be hydrolyzed during the digestion releasing the respective toxic parental compounds [1]. The transformation of masked mycotoxins to metabolites with higher toxicity than the parent compounds was also described in vitro [24,25], further highlighting the toxicological potential of the masked forms of mycotoxins (referred to as “maskedome”). Nevertheless, masked mycotoxins are not routinely screened, and this may result in an underestimation of the actual amounts of mycotoxins in foods. In this respect, Puntscher et al. [26] reported the presence of some modified forms of AOH and AME (i.e., AOH-3-glucoside, AOH-9-glucoside, AOH-3-sulfate and AME-3-sulfate) in tomato sauce samples from Italy. In particular, one sample was found contaminated not only with AOH, AME, TeA and TEN, but also with AOH-3-glucoside, AOH-3-sulfate and AME-3-sulfate. Similarly, Walravens and co-workers found tomato products (juices, sauces and concentrates) contaminated with AOH-3-sulfate and AME-3-sulfate, with a prevalence ranging from 11% to 26% and from 32% to 78%, respectively [27]. The authors reported the highest prevalence of AOH and AME in tomato sauces (86% and 78%, respectively), while ALT was most frequently detected in tomato concentrates (56%). In addition, a prevalence of TEN-contaminated products, ranging from 21% to 64% (in sauces and juices, respectively), was also reported and, interestingly, all the tested samples showed a high contamination with TeA. More recently, another study highlighted the contamination of both fresh and dried tomato samples by different *Alternaria* toxins, among which TeA was found the most frequent and abundant compound [28].

The frequent co-occurrence of multiple *Alternaria* mycotoxins was also described in many other foods, including peppers. As an example, Gambacorta and co-workers [29] analyzed samples of fresh, dried, grounded, and fried sweet pepper, wherein AOH, AME, TeA, and TEN were found together (limit of quantifications in the low µg/kg range). In particular, TeA was detected in all samples, while AOH was detected in 86%, 43%, 100% and 14% of fresh, dried, grounded and fried products, respectively. Fresh pepper samples were mostly contaminated by AME (57% of fresh pepper samples), while fried peppers were the least AME-contaminated samples (14% of fried peppers samples). ALT was detected only in 43% and 13% of fresh and grounded samples, respectively.

Beside fruits and vegetables, cereals and derived products play an important role in the exposure to *Alternaria* toxins, representing the main source of exposure for infants and toddlers [10]. According to EFSA [2], the highest mean concentrations of AOH, AME, TeA and TEN in grains were observed as follows: AOH (spelt, oats, rice); AME (oats, rice); TeA (wheat, barley, rye, spelt, oats and rice); TEN (rye). Nevertheless, in addition to the above-mentioned mycotoxins, some authors reported also the presence of other compounds in grains, although the actual co-occurrence was not clearly specified. Specifically, ragi, sorghum and spelt were found contaminated by ALT [30,31], while ATX-I was detected in spelt and wheat [20,21]. Among the least investigated mycotoxins, macrosporin, which is produced primarily by the *Stemphylium* genus but it can be produce by *Alternaria* spp. too [32], was found in corn and wheat silage [33], while ALP was detected in wheat flour samples [20]. The presence of macrosporin was also detected in dried fruits and nuts, such as almonds, dried grape berries, hazelnuts, peanuts, and pistachios [34], often in combination with other *Alternaria* mycotoxins. In a study performed by Mikušová et al. [35], dried grape berries from three Slovak winemaking regions were simultaneously contaminated by up to eight *Alternaria* mycotoxins, i.e., AOH, AME, ALT, TeA, TEN, ATX-I, ATX-II, and macrosporin, whose highest concentrations were 1308 µg/kg, 776 µg/kg, 4120 µg/kg, 159.6 µg/kg, 43.1 µg/kg, 31175 µg/kg, 624 µg/kg, and 762 µg/kg, respectively. Notably, TEN was detected in all the analyzed samples.

*Alternaria* toxins can be found also in beverages such as fruit juices, beers and wines [36,37,38,39,40,41], as well as in food supplements used for various purposes [42]. Milk thistle-based supplements for liver diseases were simultaneously contaminated by AOH, AME, TEN, and TeA with maximum concentrations of 4560 μg/kg, 3200 μg/kg, 1280 μg/kg, and 2140 μg/kg, respectively. The same mycotoxins were detected, even though at a lower concentration, in supplements used to treat menopause symptoms (containing red clover, flax seeds and soy) or for general health support (containing among others green barley, nettle, goji berries and yucca). The maximum concentration of TeA was found in supplements for general health support (6780 μg/kg), while milk thistle-based supplements showed the highest average concentrations of all mycotoxins. Notably, the beneficial effects of health-promoting compounds of food supplements might be impaired to various extents by the presence of mycotoxins. In addition, taking into account that food supplements are thought to supply specific deficiencies, the presence of mycotoxins might have a higher impact on specific categories of consumers. These aspects require urgent investigations to timely support the enforcement of specific regulations.

### 2.2. Co-Occurrence of Alternaria Toxins with Other Mycotoxins

As discussed above, many food categories may be contaminated by more than one *Alternaria* mycotoxin. However, food commodities can be simultaneously contaminated by a high number of different mycotoxins produced by molds other than *Alternaria*. In particular, mycotoxins produced by *Aspergillus*, *Fusarium,* and *Penicillium* genera frequently co-occur with *Alternaria* mycotoxins (Table 2). Among them, the most investigated and frequently detected were those produced by the genera *Fusarium* and *Aspergillus* [e.g., aflatoxins, enniatins (ENNs) and beauvericin], while the least frequently examined or detected were ochratoxins (ochratoxin A, OTA; ochratoxin B, OTB).

A study conducted by Gambacorta et al. [29] investigated the co-occurrence of 17 different mycotoxins in fresh, fried, dried or grounded sweet pepper products. Notably, all of them were contaminated by more than one mycotoxin simultaneously. In more detail, 6 out of 39 samples contained 2, 3 or 4 different mycotoxins, while the remaining samples were positive for a number of mycotoxins ranging from 5 to 16. The fried peppers showed the lowest average level of contamination (with an average mycotoxin contamination of 231 µg/kg), while the fresh pepper samples were the most contaminated (27,280 µg/kg). TeA was the most frequently detected mycotoxin (100% of samples) with an average concentration of 4817.9 µg/kg. With regard to the other *Alternaria* toxins, 93%, 56%, 33%, and 9% of pepper samples were found to be contaminated by TEN, AOH, AME and ALT, respectively. These compounds (except for ALT) were found to co-occur along with 7 other *Fusarium* mycotoxins (nivalenol, HT-2 toxin, T-2 toxin, fumonisin B_1_, fumonisin B_2_, deoxynivalenol (DON) and zearalenone (ZEN)), 4 other *Aspergillus* mycotoxins (the aflatoxins B_1_, B_2_, G_1_, and G_2_), and OTA in the most contaminated sample. It is worth mentioning the average low level of contamination of fried samples. In this respect, the frying process might have a role in lowering the content of *Alternaria* mycotoxins, though it was not directly assessed by the authors. It would be in agreement with other studies pointing to a significant reduction of mycotoxin content upon fry cooking [62]. In addition, high-temperature treatments already proved to be effective in mitigating the content of certain *Alternaria* mycotoxins [63], supporting the possible role of fry cooking in reducing the content of *Alternaria* mycotoxins. The effects of three extrusion processing parameters (moisture content, feeding rate and screw speed) on the degradation of TeA, AOH and AME in whole wheat flour have been investigated. With the optimal parameters, a reduction of 65.6, 87.9 and 94.5% was achieved for TeA, AOH and AME, respectively [63]. As a general remark, the thermal stability of *Alternaria* mycotoxins needs to be further investigated, along with the possible formation of toxic by-products, to identify effective food processing for reducing their content in food.

The co-occurrence of AOH with the *Fusarium* mycotoxins ZEN and DON and, with the ergot alkaloid ergometrine was described in beer [64]. In particular, ergometrine, a toxin produced by *Claviceps* spp. used in pharmaceutical applications [65], was detected at low concentrations in 93% of the beer samples (0.07–0.47 µg/L, median 0.15 µg/ L). AOH (0.23–1.6 µg/L, median 0.45 µg/L) and ZEN (0.35–2.0 µg/L, median 0.88 µg/L) were detected in all the beer samples, while DON was found in 75% of samples (2.2–20 µg/L, median 3.7 µg/L). In the light of the low concentrations reported above, the authors concluded that beer should not be considered among the most important source of dietary intake of AOH, ZEN and DON.

In another study, 253 samples of dried fruits and nuts were analyzed for the presence of 16 mycotoxins (aflatoxins, ochratoxins, *Alternaria* toxins and trichothecenes) [57]. The authors reported that 124 samples were contaminated with at least one mycotoxin, while more than half (66 out of 124 samples) were contaminated by at least two mycotoxins. AME was the most frequently detected mycotoxin (44/124), followed by AOH (found in 31 out of 124 samples) and enniatin B_1_ (found in 30 out of 124 samples). The most contaminated sample contained eight different mycotoxins (i.e., aflatoxins B_1_ and B_2_, enniatins B and B_1_, beauvericin (BEA), TEN, AOH, and AME). Among the number of combinations found, the most common were binary (such as BEA + AME) and tertiary (such as BEA + AME + AOH) combinations. Ochratoxin B was found occurring along with the *Alternaria* toxins AOH, AME and TEN only in two samples.

The co-occurrence of ochratoxin A with AOH and aflatoxin B_2_ was described with a low frequency in berry juice (only 1 out of 32 samples was found positive) [37]. Additionally, although 47% of berry juices were negative for all the investigated mycotoxins, at least one mycotoxin was present in 53% of the samples, with percentage distributions of 9%, 9%, 22%, and 13% for 1, 2, 3 and 4 co-occurring mycotoxins, respectively. Moreover, TEN and aflatoxin B_1_ were not detected in any of the analyzed samples, while aflatoxin B_2_ + aflatoxin G_2_ + AME + AOH and aflatoxin G_2_ + AME + AOH were the most frequently found combinations. Importantly, in 87% of the contaminated samples at least one *Alternaria* mycotoxin was detected: AOH was most frequently found (73%; concentrations from 2.5 to 85 ng/mL) followed by AME (67%; concentrations from 267 to 308 ng/mL). Similarly, the co-occurrence of *Alternaria* toxins with other mycotoxins was also reported in dried fruit samples from China (apricots, raisins, dates, and wolfberries) [58]. In particular, 64.6% of the samples were contaminated by at least one mycotoxin, while 31.4% of the samples were contaminated with two to four compounds. TeA was the most abundant (from 6.9 to 5665.3 µg/kg) and frequently detected compound, followed by TEN (20.5% of samples) and mycophenolic acid (MPA; 19.5% of samples). MPA is produced by various *Penicillium* species and it is used as an immunosuppressant drug to prevent organ rejection after transplantation. In terms of safety, its occurrence in food may raise concern on account of its potential to predispose susceptible individuals to infectious diseases [58]. The combinations TeA + TEN and TeA + MPA were found with a prevalence of 13.2% and 11.4%, respectively [58]. In addition, TeA was simultaneously detected along with OTA in 7% of samples, with an apparently inverse relationship: the higher the concentration of TeA, the lower the concentration of OTA. This might be due to competition phenomena between mycotoxin-producing fungi or due to degrading processes, as reported by Müller et al. [66]. They described an inverse correlation between the increase of AOH, AME and TeA production and the decrease of *Fusarium* toxins (DON and ZEN) possibly due to the degradation of the latter by *Alternaria* strains. In this context, in vitro studies on the synthesis of mycotoxins during the co-incubation of *Alternaria* strains with other fungi may be useful to investigate the existence of a possible mutual influence, which seems likely to exist on the basis of low level of co-occurring mycotoxins reported so far in the literature.

As already reported in Section 2.1, food supplements might be highly contaminated by *Alternaria* toxins. However, *Alternaria* toxins can be found in food supplements also along with other mycotoxins. As an example, Veprikova and co-workers found 66 out of 69 samples contaminated by more than one mycotoxin. Specifically, 58% of milk thistle-based supplements contained more than 12 different mycotoxins simultaneously, while one of the most contaminated samples contained 14 different mycotoxins, i.e., AOH, AME, TEN, 3-acetyl-DON, beauvericin, fusarenon-X, ZEN, HT-2 toxin, T-2 toxin and enniatins B, B_1_, A and A_1_ [42]. The most common combinations described were ENNs + HT-2/T-2 + AOH + AME + TEN and ENNs + AOH + AME + TEN + MPA. As a general remark, the state-of the-art of food supplements contamination warns about a potentially dangerous scenario. Indeed, although to date no maximum limits of *Alternaria* mycotoxins have been defined for food, the relatively high concentrations of mycotoxins occasionally detected in food supplements might suggest the need to perform dedicated risk assessment studies. Therefore, further occurrence and exposure studies have to be done urgently paving the ground to timely enact specific regulations for food supplements.

## 3. Individual Toxicity of Main *Alternaria* Toxins and Combined Toxicity with Other Mycotoxins and Bioactive Compounds of Food Origin

*Alternaria* species may produce a huge variety of different mycotoxins showing a great variability in terms of chemical structures [68]. AOH, AME, TeA, ALT, and altertoxins (I, II, III) are considered the most relevant for food toxicology, taking into account their occurrence and/or toxicity. Nevertheless, in vivo toxicological data currently available are not adequate for a proper risk assessment and, therefore, they are not sufficient to define toxicological standard values for the establishment of maximum limits in food and feed. At present, the only LD_50_ values are available, even if they refer to a limited number of compounds (Table 3).

As a general remark, except for these few mycotoxins, very few data are available for the other members of the *Alternaria* mycotoxin family, which still remain largely uncharacterized in terms of toxicity and mechanisms of action.

As already discussed, the simultaneous occurrence of more than one *Alternaria* mycotoxin, also in combination with other mycotoxins produced by different fungi, is common in food. In this respect, it is important to remark that the risk assessment of mycotoxins currently relies on single substance effects [2,74], neglecting any possible mutual combined actions due to simultaneous exposure. These mycotoxin-mycotoxin interactions might modify the individual toxicity of compounds, likely resulting in a final toxic outcome different from the single compound tested alone. In addition, it must be considered that many extra-nutritional constituents (such as bioactive food constituents) are widely present in food, and their biological activity may also interfere with mycotoxin activity. The combined actions can be referred to as: (i) additive effects, when the final toxicity is the sum of the individual toxic effects of compounds; (ii) synergistic effects, when the resulting total toxicity is greater than the sum of individual effects or iii) antagonistic effects, when the opposite is the case and the combinatory effect is less than additive [75]. Several mathematical models and methods are commonly used to evaluate the nature of the combined effects of toxic compounds. Among them, the most common are the independent joint action model and the combination index-isobologram method. The first one allows to calculate an expected additive value from the effects of the single compounds [76] that can in turn be compared to a measured combinatory effect. The combination index-isobologram method allows to take into account the shape of dose-response curves when determining the type of interaction (synergism, additive effect and antagonism) [75,77]. This is considered the state-of-the-art model; however it can be challenging to meet the requirements to apply it.

As shown below, the evidence collected so far clearly states that synergistic effects of mycotoxins in mixtures with other compounds (either mycotoxins or other food components) may have important consequences on the single-compound activity. This might have an impact on the assessment of risk related to the presence of *Alternaria* mycotoxins in food, which should consider the mixtures, rather than focusing on single-compound evidences. The individual toxicity of the main *Alternaria* mycotoxins and the effects of their combination with other mycotoxins or food constituents are reported in the following sections.

### 3.1. Individual Toxicity of Alternaria Mycotoxins

#### 3.1.1. Genotoxic Effects

Among the best characterized *Alternaria* toxins, those with genotoxic properties are considered of most concern for human health by regulatory authorities. This particularly applies to AOH and AME, for which the EFSA concluded that “the estimated mean chronic dietary exposures at the upper bound and 95th percentile dietary exposures exceeded the TTC value” in their latest exposure assessment [10], and thus called for more data regarding exposure and toxicity of those metabolites [13].

In human cells, both AOH and AME have been reported to induce DNA strand breaks in the comet assay at concentrations ≥1 µM [78], to act clastogenic at ≥2.5 µM [79] and to possess mutagenic potential at ≥10 µM, as measured by HPRT and TK gene mutation assays [80]. An in vivo study on mice did not find AOH to cause systemic DNA damages in liver tissue and bone marrow [81]. However, the authors argue that any toxicity of the substance would probably be limited to the gastrointestinal tract due to poor bioavailability, but did not include corresponding organs in their survey.

Concerning the mechanisms of action, both AOH and AME were found to act as a topoisomerase (TOP) poison at micromolar concentrations, affecting the activity of both TOP I and TOP II, with a certain preference for the α isoform of TOP II [78]. Those enzymes are needed to untangle the DNA for replication or transcription, a process which involves the induction of a transient DNA strand break that is re-ligated at the end of the catalytic cycle. Poisoning of these enzymes by small molecules results in a toxin-dependent stabilization of the covalent DNA–topoisomerase complex (i.e., the so-called “cleavable complex”). Stabilization of the cleavable complex by TOP “poisons” hinders release of TOP in the catalytic cycle and re-ligation of the DNA, thus resulting in a persistence of the initially induced strand break. Thus, TOP poisons are commonly described to act genotoxic [82].

An additional mechanism contributing to the toxicity of *Alternaria* toxins is the induction of intracellular reactive oxygen species (ROS), which indicate oxidative stress. ROS production induced by AOH and AME might play an important role in the inhibitory effects on cell proliferation observed in different cellular models [83,84].

Of note, ALT and iso-ALT were not found to affect topoisomerase activity [78], probably due to their less planar structure not allowing for DNA intercalation in comparison to AOH/AME [85].

However, it was observed that extracts from cultured *Alternaria* strains by far exceeded the genotoxicity of their dibenzo-α-pyrone contents [86]. This has led to the discovery of the epoxide-carrying perylene quinone ATX-II as a major contributing factor to the genotoxicity of naturally occurring mixtures of *Alternaria* toxins [87,88]. Later on, not only ATX II, but also the structurally related STTX-III was found to be more mutagenic then AOH. Regarding their mode of action, these mycotoxins were also found to act as inhibitors of TOPs at high concentrations. However, their main genotoxic mode of action is thought to be the formation of DNA adducts, a hypothesis which still awaits experimental confirmation [87,88,89].

Of note, there is speculation that yet not characterized secondary metabolites might also possess genotoxic properties, as an *Alternaria* extract very low on dibenzo-α-pyrones, which was additionally stripped off ATX-II and STTX-III, still maintained substantial DNA-damaging properties [90].

#### 3.1.2. Endocrine-Modulating and Other Toxic Effects

AOH and AME, as well as other related metabolites, were reported to elicit estrogenic effects in cellular systems. In particular, AOH was described to be able to activate both ER-α and β but with a greater affinity (approximately ten-fold higher) for ER-β [79,91], although the binding strength is 10,000-fold weaker than the endogenous hormone estradiol. AME was found to be slightly more potent than AOH at 10 µM, and the methylation at the 9-OH group was thought to improve the molecular fitting within the estrogen receptor pocket [92]. AOH was additionally found to induce androgenic effects in the yeast androgen bioassay [93]. Recently, computational studies reported that mutations of the androgen receptors might affect the capability of AOH to bind and possibly stimulate the activation of receptors [94]. Moreover, increases in progesterone and estradiol levels, as well as in progesterone receptor expression, were reported in human adrenocarcinoma H295R cells treated with AOH, supporting its actual role as endocrine disruptor [95]. However, in naturally occurring mixtures of *Alternaria* toxins, endocrine-disrupting effects of AOH and related metabolites might be “quenched” by cytotoxic and anti-estrogenic properties of co-occurring compounds, as recently demonstrated in Ishikawa cells [90].

In addition to the above listed toxic effects, AOH and AME were found to modulate innate immunity in both human bronchial epithelial BEAS-2B cells and mouse macrophages RAW264.7, through the suppression of the lipopolysaccharide-induced innate immune responses [96]. This activity was also confirmed in THP-1 derived macrophages by Kollarova et al. [97]: AOH, in fact, suppressed lipopolysaccharide (LPS)-induced NF-κB pathway activation, induced transcription of the anti-inflammatory cytokine IL-10, and reduced the transcription of the pro-inflammatory cytokines IL-8, IL-6 and TNF-α.

TeA deserves a particular mention as, unlike the other *Alternaria* mycotoxins, it exerts toxic effects mainly by inhibiting the release of proteins from the ribosome. Although a low toxicity of this mycotoxin has been reported in vitro [86,98], in vivo studies carried out on several animal models highlighted more severe effects such as emesis, tachycardia and haemorrhages [18].

### 3.2. Combinatory Effects of Alternaria Mycotoxins

There are only a few studies investigating the combinatory effects of *Alternaria* mycotoxins, though food may be quite often simultaneously contaminated by more than one single compound as shown, for instance, for AOH and AME (Section 2.1). Notably, these two mycotoxins are not of particular concern in terms of cytotoxic effects, also on account of the high concentrations required to cause harmful effects when tested individually. However, the simultaneous exposure to AOH and AME may have significant effects on the overall toxicity in respect to their individual testing. In more detail, their combined effects (1:1 concentration ratio) were invested by Bensassi et al. on the human intestinal cell line HCT-116 [99]. No significant difference in cell viability was detected at 25 µM up to 24 h of exposure when mycotoxins were tested either individually or in combination. Conversely, both mycotoxins reduced cell viability about 30% after 24 h of exposure when tested individually, while they reduced viability about 50% when tested in combination. In this study, the nature of interactive effects was described to be additive, while Fernández-Blanco and co-workers reported synergistic effects in Caco-2 cells after 24 h of exposure to AOH and AME in a 1:1 binary combination and in a concentration range from 3.125 to 30 µM [83]. Moreover, the AOH-AME binary combination reduced cell proliferation to a greater extent than AOH alone at all tested concentrations, while it had greater effects than AME alone at 15 and 30 µM. The binary mixture also caused a greater dose-dependent reduction of cell proliferation after 48 h of incubation (in the concentration range 7.5–30 µM) than AOH or AME tested alone. In this case, the nature of the interactive effects was described as synergistic or additive at small or higher fraction affected, respectively.

The effects exerted by the simultaneous exposure to AOH and the genotoxic *Alternaria* mycotoxin ATX-II were investigated by Vejdovszky et al. [100] on two intestinal (HT-29, HCEC-1CT) and one hepatic (HepG2) cell line. Seven different concentrations, ranging from 500 nM to 10 µM for ATX-II and from 5 µM to 100 µM for AOH were tested for binary combinations (constant ratio of 1:10, ATX-II:AOH). As a result, the HT-29 cell line was found to be the least sensitive to cytotoxic effects mediated by the two tested mycotoxins and significant differences in cell viability were found starting from the combination 5 µM:50 µM (ATX-II:AOH). Among the different concentrations tested, the highest decrease in cell viability observed was of nearly 40%. Notably, the cell treatment at low mycotoxin concentrations led to an increase in mitochondrial activity in the co-treated samples. HepG2 cells were found the most sensitive to the cytotoxic effects exerted by AOH, while HCEC-1CT cells proved to be the most sensitive to the effects of ATX-II. Combining these two mycotoxins, an increased sensitivity to cytotoxic effects was also found in the HepG2 cell line, leading to a reduction in cell viability starting from the combination 1 µM:10 µM (ATX-II:AOH). Although most of the tested 1:10 combinations showed additive effects, antagonistic effects were reported in HCEC-1CT and HepG2 cell lines, while only one of the combinations analyzed showed synergistic effects on HepG2 cell line (750 nM ATX-II:750 nM AOH, 1:1 ratio). Modifications of microRNAs expression profile after incubation of HepG2 cells with the mixture 10 µM AOH:1 µM ATX-II may partially explains such effects. The combined exposure caused a significant increase of miR-224 expression after 12 h of exposure, which was no longer over-expressed after 24 h, while miR-192 and miR-29a were respectively down-regulated and up-regulated after 24 h. In addition, miR-29a was up-regulated also in samples treated with AOH alone, suggesting a possible role in the up-regulation of this miRNA by the binary mixture. Interestingly, these three microRNAs are involved in the regulation of apoptotic processes and the observed modifications led the authors to conclude that such miRNAs may be in part involved in the antagonistic effects observed for some of the combinations tested.

As previously described, *Alternaria* mycotoxins are often found in food commodities along with *Fusarium* mycotoxins. In a recent study [101], the cytotoxic effects and the type of interactions of AOH combined with *Fusarium* mycotoxins enniatin B and DON were evaluated after 24, 48, and 72 h of exposure in Caco-2 cells. For binary and tertiary combinations, five different concentrations, ranging from 0.3125 to 5 µM for enniatin B and DON, and from 1.875 to 30 µM for AOH, were tested. The binary combinations enniatin B + AOH (1:6 ratio) led to higher cytotoxic effects compared to AOH tested alone at all the timepoints and concentrations tested. However, no difference between enniatin B tested alone and in mixture was observed, suggesting that the cytotoxic effects were mainly mediated by enniatin B. With regard to the binary combinations DON + AOH (1:6 ratio), the resulting cytotoxicity after 24 h of exposure was lower than that exerted by DON tested alone. On the contrary, an opposite trend was observed after 48 and 72 h of exposure. As expected, the tertiary mixtures enniatin B + DON + AOH (ratio 1:1:6) led to a greater decrease, albeit of slight intensity, of cell viability compared to the binary combinations. Although the pattern was not uniform along the fraction affected, the application of the isobologram analysis described the interactions in the binary mixtures as additive and synergistic, depending on the concentrations and timepoints tested. Interestingly, the ternary combinations showed antagonistic effects, which were described as due to competition mechanisms at the same receptor site. In this respect, it is worth mentioning the marked diversity of these mycotoxins in terms of chemical structures. Taking into account that the competition to the same protein site usually requires strict conservation of key structural motifs [102], the inherent structural heterogeneity among enniatin B, DON and AOH is not fully compatible with their capability to physically compete with the same site. Therefore, both the molecular mechanisms and the network of biological targets involved in such antagonistic behavior need to be precisely described to better understand the effects of the enniatin B/DON/AOH ternary combination.

The effects of binary and ternary combinations of AOH with the DON’s acetylated derivatives 3-ADON and 15-ADON were also investigated on HepG2 cells up to 72 h of incubation [103]. Constant ratios of 16:1 (AOH: 3-acetyl-ADON and AOH:15-acetyl-DON) and 16:1:1 (AOH:3-acetyl-DON:15-acetyl-DON) were chosen to test these mixtures, with concentrations ranging from 3.2µM to 24µM for AOH, and from 0.2 µM to 1.5 µM for DON’s derivatives. Cytotoxicity ranking was found to be the same for all tested time points (AOH+3-acetyl-DON + 15-acetyl-DON > AOH + 3-acetyl-DON > AOH + 15-acetyl-DON) and a concentration-dependent decrease in HepG2 cell viability was found in all tested mixtures. The effects caused by binary and ternary mixtures were described to be mainly synergistic, but some exceptions were found for AOH + 3-acetyl-DON at 72 h (where additive effects were observed at higher fraction affected), and for AOH + 15-acetyl-DON (where additive or antagonistic effects were observed depending on the concentration and timepoint tested).

Binary effects of TeA with the *Fusarium* mycotoxins enniatin B, ZEN, DON, nivalenol and aurofusarin (AURO) were also evaluated on Caco-2 cells with two different concentration sets, named “low concentrations” (none or slight cytotoxic effect) and “high concentrations” (pronounced cytotoxic effect) [104]. TeA combinations at “low concentrations” of mycotoxins did not show significant differences between the measured and expected effects (calculated on the basis of the Independent Joint Action model). This indicates that the combinations of TeA at “low concentrations of mycotoxins” only determined additive effects. On the contrary, binary combinations at “high concentrations” led to lower cytotoxic effects then the calculated additive effects. Additional investigations allowed getting more details about the type of interactions between TeA and *Fusarium* mycotoxins. No difference in cytotoxicity was found in samples co-treated with enniatin B and ZEN keeping the concentration of *Fusarium* mycotoxins constant (from 5 to 50 μM depending on the mycotoxin) and varying that of TeA (from 1 µM to 250 µM). Indeed, the cytotoxicity of binary mixtures with TeA was found to be equivalent to the toxicity of toxins tested individually. Notably, the toxic effect induced by 10μM DON was reduced in a concentration-independent manner by the combination with TeA at concentrations between 10 μM and 200 μM. A similar trend was found for the combination with 10 μM nivalenol, although differences were not statistically significant. Keeping in mind that TeA and the *Fusarium* mycotoxins DON and nivalenol are known to inhibit protein synthesis in vitro [104], the lower cytotoxic effects of binary mixtures might be due to a molecular interplay at the level of protein synthesis inhibition. Nevertheless, considering that nivalenol and DON inhibit protein synthesis by different mechanisms (i.e., by inhibiting the initiation or elongation-termination steps, respectively) [105], the observed effects cannot be straightforwardly explained in terms of mechanisms of action pointing out the need of investigating further the molecular basis of such interaction. In this respect, the inhibition of protein synthesis by TeA may modify the expression of specific factors, including metabolizing enzymes, and consequences on the pattern of metabolites produced by cells are thought likely. This is of particular relevance as some trichothecenes metabolites might be involved in mediating ribotoxic effects of parent mycotoxins, as supported recently by in silico studies [106]. On this basis, TeA might have indirect effects on trichothecenes toxicity acting on their metabolism and changing the relative abundance of ribotoxic metabolites produced.

Recently, an interesting study was performed by Solhaug et al. that investigated the ability of AOH, DON and ZEN in binary and tertiary mixtures to affect immune response checking the differentiation of monocytes to macrophages [107]. The differentiation process leads to several changes, including modifications of the expression of some cell surface markers such as CD14, CD11b and CD71. AOH, DON and ZEN were able to modify the expression of these markers in THP-1 monocytes, but with some differences: while AOH affected the expression of the all set of markers, DON did not modify the expression of CD71 and ZEN altered only the expression of CD-14. Since CD-14 was the only marker modified by all the three mycotoxins, its expression was used to evaluate the type of interactions in binary and ternary mycotoxins mixtures by applying the “Concentration Addition” (CA) and the “Independent Joint Action” (IA) models. Since authors did not find significant differences between the experimental data and the predicted models, the type of interaction was described to be additive. Remarkably, at the lowest concentrations of the AOH + ZEN combination, the confidence interval of the predicted CA model did not overlap with the experimental values, suggesting a possible synergistic effect. The same results were obtained for the binary combinations through the application of the isobologram analysis. To verify if the observed inhibitory effects of AOH, DON, and ZEN on the up-regulation of CD14 led to a real reduction in macrophage activation, the pro-inflammatory cytokine TNFα and its gene expression were quantified after incubation with single mycotoxins. Contrary to what was observed for AOH and ZEN, DON induced an increased secretion of TNFα following the increase of TNFα gene expression, in spite of its inhibitory action on the up-regulation of CD14. The expression of NF-kB, a protein complex involved in TNFα expression, might provide a plausible explanation to these differences. Indeed, ZEN was reported to reduce the expression of NF-kB [108], and, recently, also AOH showed the ability to suppress the lipopolysaccharide-induced NF-kB pathway activation, resulting in the reduction of TNFα [97]. In contrast, DON was found to induce both NF-kB activation and TNF-α expression, but the signaling pathway was different from those activated by ZEN and AOH [109].

### 3.3. Combined Effects with Bioactive Food Constituents

Beside the combined action of the different members of *Alternaria* mycotoxins group, also in combination with mycotoxins produced by fungi other than *Alternaria*, it is important to take into consideration even the complex interactions that these mycotoxins may have with the other bioactive compounds of food origin.

In this contest, Vejdovszky et al. recently investigated the combinatory estrogenic effects of the isoflavone genistein (GEN) in combination with ZEN and AOH [110]. To elucidate the combinatory effects, the human endometrial adenocarcinoma Ishikawa cell line was chosen as a model system and the phosphatase alkaline (ALP) activity assay was used to measure estrogen receptor activation. The xenoestrogens under investigation were tested at different concentrations (ranging from pM to µM) after 48 h of incubation. All of them increased the ALP activation when tested individually, with the following order of potency in terms of EC50: E2 (17β-estradiol; used as positive control) > ZEN > GEN > AOH. Moreover, these xenoestrogens did not only differ in terms of potency, but also in terms of efficacy as none of them (at any concentration) was able to determine the same effects induced by 1 nM E2. A possible explanation for this finding is that AOH, ZEN and GEN might act as partial agonists. The lower capability to satisfy the pharmacophoric requirements of estrogen receptors pockets in comparison to E2 [111,112] might provide a structural rational to explain such evidence. With regards to binary mixtures of GEN with ZEN or AOH, some of them resulted in significantly higher effects than the respective compounds tested individually, clearly pointing out the existence of synergistic effects. However, combinations of GEN-AOH activated ALP to a lower extent than ZEN-AOH mixtures. It must be highlighted that in many studies ZEN was found to be more estrogenic than AOH, and this could partly justify the lowering of estrogenic effect observed in combinations [110]. In addition, while the authors noted the preference of AOH and GEN to ERβ, ZEN was previously described with a higher affinity for ERα [113]. The simultaneous activation of both α and β estrogen receptor isoforms in the ZEN-GEN and ZEN-AOH binary mixtures may explain the stronger synergistic effects observed. Although some GEN-AOH combinations showed synergistic effects, other combinations at very low doses led to antagonistic effects. Indeed, anti-estrogenic effects were found testing the combination 0.001 μM GEN-0.1 μM AOH and observing a reduction of ALP activation (10.9%) compared to the control (vehicle). A subsequent more-in-depth analysis of the combinatory effects, performed through the combination index and the isobologram method, allowed to determine the type of interactions occurring in the different combinations. Both methods showed that the combinatory effects of GEN and ZEN in the constant ratio of 1000:1 were mainly synergistic and, only at very low or very high effect levels, additive or antagonistic effects were observed. In the constant ratios of 100:1 and 10:1, the substances led to a strong antagonism at low effect levels, and to a strong synergism at higher effects. Comparable outcomes were reported for the 1:10 GEN:AOH ratio (which showed antagonistic or synergistic effects at low or high effect levels, respectively), while the 1:5 combination ratio determined mainly antagonistic effects. Additionally, the 1:1 GEN:AOH ratio resulted in the onset of synergistic effects up to about 65% of the maximum ALP activation observed (E2 1nM). Above, additive or antagonistic effects were observed depending on the concentrations tested. Thus, the nature of the interactions seemed to depend on both the ratio of substances and the specific concentrations tested.

It was also established that AOH is able to cause oxidative stress and to exert genotoxic effects in different cellular models, mainly by acting as a topoisomerase poison [78]. Aichinger et al. investigated the effects of AOH in combination with the two polyphenols GEN and delphinidin (DEL) [114]. These two compounds are known for their antioxidant effects at specific concentrations, although pro-oxidant effects at certain concentrations were also demonstrated [115,116]. Both GEN and DEL were found to interact, albeit with different mechanisms, with topoisomerases: while GEN usually acts as a topoisomerase poison, turning the enzyme into a DNA-damaging agent, DEL acts as a catalytic inhibitor of topoisomerase hindering the formation of the TOP-DNA intermediate. Therefore, considering both the antioxidant effects and the interaction with the topoisomerases, a modification of the effects induced by AOH may be expected when the mycotoxin is combined with these two polyphenols. Preliminary investigations on the combinatory cytotoxic effects were conducted in HT-29 colon carcinoma cells with concentrations ranging from 1 to 100 µM (1:1 ratio): cytotoxicity was observed starting from 25 µM for AOH and GEN, and from 50 µM for DEL. Both AOH/GEN and AOH/DEL combinations led to cytotoxic effects starting from 25 µM (1:1 ratio) and the type of interactions was described as synergistic, with a tendency to lose synergism when increasing cytotoxic effects. DNA strand breaks and oxidative DNA damages of the combinations of AOH (50 µM) with DEL (10–100 µM) or GEN (25–250 µM) were evaluated by performing an alkaline comet assay with or without treatment with formamidopyrimidin-DNA-glycosylase (FPG). When combined, DEL and AOH showed marked antagonistic effects at 50 µM in the FPG-untreated samples, while lower oxidative DNA damages were observed at 25 and 100 µM. Similar results were found for the combination AOH/GEN at 25 and 100 µM, which showed a lower oxidative damage than AOH tested individually. The authors also evaluated the influence of the co-incubations on the stabilization of the topoisomerases/DNA intermediate (the so-called “cleavable complexes”), which is typically due to the action of topoisomerase poisons (such as AOH). The AOH/GEN combination did not increase the formation of cleavable complexes, rather an antagonistic effect was found at the highest GEN concentration tested (100 μM). Antagonistic effects were also found in AOH/DEL combinations starting from 25 µM. These results were partially attributed to the dual anti-oxidant or pro-oxidant properties of the polyphenols. In this respect, simultaneous short-time incubations with AOH and DEL led to a reduction of AOH-induced ROS generation at concentrations of DEL starting from 1μM. On the contrary, GEN induced oxidative stress per se and did not suppress the pro-oxidative effects induced by AOH. Moreover, 24-h pre-incubations with polyphenols followed by incubation with AOH, did not result in any change in pro-oxidant effects of AOH. This evidence led to exclude any possible modulations of anti-oxidant defense systems as a mechanism underlying the observed antagonistic effects. Therefore, direct anti- or pro-oxidant activities are reasonably as the base of the effects observed during the co-incubations with DEL and GEN. On this basis, DEL could help in preventing the genotoxic effects of AOH, but, considering the low systemic bioavailability of DEL, these protective effects may be limited to the gastrointestinal tract only [117,118].

The same authors also investigated the effects of DEL in combination with ATX-II, one of the most genotoxic *Alternaria* toxins [119]. As reported for the combination with AOH, DEL reduced both DNA strand breaks and oxidative damage in HT-29 cells after short-time co-incubation with ATX-II. The type of interaction was found to be antagonistic according to the applied “independent joint action” model. The production of ROS induced by 10 μM ATX-II was also reduced by DEL in concentrations from 1 μM to 100 µM, but these reductions cannot fully explain the huge reduction of genotoxic effects observed following the co-incubation with DEL. Indeed, no increase of ROS production was observed at the concentration of ATX-II used in the comet assay (1 μM). In cell-free conditions, a reduction of the concentration of ATX-II was found upon co-incubation with DEL. The authors suggested that DEL, after being degraded to phloroglucinol aldehyde (PGA) and gallic acid (GA), might react with ATX-II neutralizing its epoxy group, which is the reactive chemical moiety presumably responsible for genotoxicity. Considering the hypothesis that PGA can react with ATX-II, it is important to underline that the reduction of adverse effects of this mycotoxin may actually occur in subjects that follow diets with a high content of anthocyanins as they are prone to release PGA during digestion.

In the context of the evaluation of combinatory effects between *Alternaria* toxins and bioactive compounds of food origin, polyphenols represent a class of compounds of great interest since they are widely distributed in those food categories which are prone to contamination with *Alternaria* mycotoxins. Quercetin (QUE) is one of the most abundant flavonoids in human diets. QUE has been previously associated to several potential health benefits mainly related to its antioxidant properties, although pro-oxidant effects at certain concentrations have also been described [120]. The potential ability of QUE to reduce the cytotoxicity of AOH and AME was investigated by Fernàndez-Blanco et al. [83]. Although cytoprotective effects were attributed to QUE [121], simultaneous exposure of Caco-2 cells to AOH and QUE (at concentrations ranging from 3.125 to 100 μM) did not result in any cytoprotective effect. In particular, no significant differences were found between the QUE-AOH combination and AOH tested alone after 48 h of exposure. However, the combination significantly affected cell viability at 24 h of treatment in comparison to AOH tested alone. Similarly, no difference between the binary combinations QUE + AME and AME tested alone were detected and, additionally, no cytoprotective effect was found in the tertiary combination AOH + AME + QUE at any of the tested concentrations. Therefore, QUE was not effective in reducing the effects of AOH and AME.

Possible cytoprotective properties of food components against the effects of AOH were also evaluated by Vila-Donat et al. in Caco-2 cells [122]. Keeping in mind that AOH may contaminate legumes (including soybeans and lentils), the authors investigated the effects of AOH in combination with soy saponin I (Ss-I), which was previously found to possess antioxidant activity, or with a lentils extract. In particular, the authors used two different approaches to evaluate the effects of Ss-I and lentil extract: (i) the first one consisted in pre-treating cells with Ss-I (6.25 µM) or with the extract, and then refreshing the growth medium and testing different dilutions of AOH (ranging from 3.125 to 50 μM); (ii) the second approach aimed at evaluating the combinatory effects and the type of interaction co-incubating AOH with Ss-I or lentil extract. By using the first approach, no differences were found between samples pre-treated with Ss-I and samples treated only with AOH. As an exception, the highest AOH concentration tested (50 μM) caused an increase in cell viability in the pre-treated samples. In contrast, co-treatments with AOH + Ss-I (1:1 ratio) above 6.25 µM resulted in an increase in cell viability compared to AOH tested alone. These results suggested that Ss-I likely acted via a direct interaction rather than modulating intracellular defense systems. With regard to cytoprotective effects of the lentils extract, only one single combination was tested and about 30% increase in cell viability was found in comparison to AOH tested alone.

## 4. The Key Role of Bioactive Compounds

Food is a complex matrix composed by macro- and micro-nutrients, containing also a huge number of non-nutrient compounds that may exert several biological activities. These compounds can interfere at different levels with mycotoxin activities. For instance, they can: (i) activate or inhibit enzymes involved in the metabolism of xenobiotics; (ii) act as anti-oxidant or pro-oxidant compounds; (iii) act as receptor agonists or antagonists targeting, in some cases, the same biological targets of mycotoxins; (iv) modify the expression of genes encoding proteins involved in the regulation of important physiological functions. On this basis, bioactive compounds of food origin may determine the onset of additive, synergistic or antagonistic effects when combined with *Alternaria* mycotoxins. Keeping in mind that the application of mitigating strategies along the food chain are supposed to progressively reduce the dietary exposure to toxicants, the assessment of combinatory effects of mycotoxins with other food constituents will be the most accurate and realistic, but also highly challenging tasks to achieve in the next decades. The challenge will be even harsher taking into account that many food constituents potentially interplaying with mycotoxins are generally recognized as health promoting (i.e., polyphenols) and the consumption of foods rich in such compounds is typically recommended in healthy diet habits. In this framework, this section focuses on the modulation of *Alternaria* mycotoxins toxicity by bioactive compounds.

One of the best characterized toxicological endpoints of *Alternaria* mycotoxins likely affected by food constituents is the estrogenic activity. As a matter of fact, estrogenic and anti-estrogenic effects of bioactive compounds might markedly modify the overall estrogenicity of the *Alternaria* mycotoxins AOH and AME. In terms of risk characterization, this might change the toxicological relevance of such mycotoxins case by case, though they show a weak estrogenicity per se, depending on the composition of chemical mixtures in given foods. In this respect, foods prone to *Alternaria* contamination with a high content of potentially interfering constituents (e.g., polyphenolic phytoestrogens) are legumes (especially soy) and some alcoholic beverages (especially wine and beer). In particular, soybeans and derived products are among the richest dietary sources of phytoestrogens, and many of the isoflavones of soy (including genistein, daidzein, glycitein, and coumestrol) induce estrogen-receptor dependent estrogenic stimuli [123]. As a matter of fact, combinations of GEN-AOH at specific concentrations have been demonstrated to determine synergistic or antagonistic effects in Ishikawa cell line [110]. Similarly to soybeans, hops used to produce beer is characterized by the presence of some prenylflavonoids (e.g., naringenin, 8-prenylnaringenin, 6-prenylnaringenin, 6,8-diprenylnaringenin, and 8-geranylnaringenin) that are potent phytoestrogens with a dual effect being able to bind both estrogen receptor isoforms and to inhibit specific enzymes involved in the estrogenic cellular responses [124,125]. In this context, Aichinger et al. [126] demonstrated the ability of the phytoestrogens from hops xanthohumol and 8-prenylnaringenin to antagonize the estrogenic effects of the *Fusarium* mycotoxins ZEN and α-ZEL. Therefore, possible interactions can be expected also in combination with the estrogenic *Alternaria* toxins AOH and AME. Other important food constituents able to modulate estrogen receptor activity are resveratrol and β-sitosterol, whose primary dietary sources are peanuts, grapes, and wine. Resveratrol, in particular, may exhibit a super-agonist activity inducing a stimulation higher than the endogenous ligand 17β-estradiol in estrogenic gene report assay, even if anti-estrogenic effects were found in the MCF-7 cell line [127]. Although evidences have been not yet collected, these compounds are likely to affect the estrogenicity of *Alternaria* mycotoxins.

Another focal point of the cross-talk between mycotoxins and food components that requires further investigations is the modulation of the aryl hydrocarbon receptor (AhR) [128]. The cascade of events following the activation of AhR is of particular interest in toxicological investigations as it modulates the expression of genes involved in detoxification and transport of various xenobiotics, including the expression of cytochrome P450 family members. Interestingly, AOH and AME were able to bind and activate AhR, causing the increase of CYP1A1 expression and promoting their own metabolism [129]. This process was not affecting the mycotoxin-dependent production of ROS in murine hepatoma cells (Hepa1c1c7). In addition, the authors showed that mycotoxins reduced the number of cells via an AhR-independent process, although the apoptotic phenotype was found only in cells with functional AhR and ARNT [129]. With regard to the ability of AOH to suppress the lipopolysaccharide-induced inflammation previously mentioned, Grover & Lawrence did not find any correlation between AOH-mediated AhR activation and the suppression of the inflammation found in BEAS-2B cells [96]. Thus, despite the increased metabolism of AOH and AME, AhR activation does not seem to raise much concern for ROS production, cytotoxic and immunosuppressive effects, further studies are needed to determine the toxicity of hydroxylated metabolites (e.g., estrogenic properties). In particular, Dellafiora and co-workers showed that hydroxylated forms of AOH and AME cannot interact with estrogen receptors in vitro, pointing to the relevance of phase-I metabolism to modify the toxicodynamic of these mycotoxins. However, methylation of respective catecholic metabolites might reactivate the estrogenic potential [92].

Besides AOH and AME, many food constituents have been described to activate or inhibit AhR. Thus, they are likely to interfere with the ability of AOH and AME to bind AhR and/or with the metabolic processes following the activation of AhR. Foods consumed worldwide such as potatoes, cruciferous, bread, hamburgers, and citrus juices were investigated for the presence of natural AhR-agonists (NAhRAs) [128]. Among these, indole-3-carbinol, and many polyphenols and furocoumarins were found to be responsible for the activities shown by cruciferous vegetables (Brussels sprouts, broccoli, cabbage) and citrus juices, respectively. On the contrary, the activation of AhR induced by the baked or fried foods tested is thought due to secondary chemicals originating from the high-temperature processing, such as polycyclic aromatic hydrocarbons (PAHs), heterocyclic amines or Maillard products [128]. In addition, many dietary flavonoids showed a significant context–dependent AhR agonist or antagonist activities, depending on the concentration and cell types tested [130]. As an example, galangin, GEN, daidzein, and diosmin were found to be AhR agonist only in Hepa-1 cells, while cantharidin acted as an agonist only in human HepG2 and MCF-7 cells. On the contrary, AhR antagonist activities were shown both in MCF-7 and HepG2 cells by luteolin, while the antagonistic activity of kaempferol, quercetin and myricetin was strictly dependent on the cell context [130]. Many other flavones, flavonols, flavanones, isoflavones, and catechins also showed a high affinity to the AhR at dietary exposure levels [131]: apigenin, luteolin, quercetin, kaempferol, and myricetin were found to inhibit the activation of AhR induced by the most potent AhR activator identified so far (2,3,7,8-tetrachlorodibenzo-*p*-dioxin at 5 nM in MCF-7 cells) [131]. Taken together, these findings suggest that the AhR-dependent effects of food constituents strongly depend on both the chemical environment (which may significantly change among the different type of food) and on the cell type tested. Therefore, both the metabolism of *Alternaria* mycotoxins in vivo and their ability to modulate AhR could change depending on the food-specific chemical mixture.

An additional noteworthy activity of AOH and AME common with a number of food components is the capability to poison topoisomerases. In particular, many food bioactives naturally occurring in fruits, vegetables and legumes have been shown to affect the activity of both topoisomerase I and II. Taking into consideration that some *Alternaria* mycotoxins exert important genotoxic effects via either the inhibition or poisoning of these enzyme (see Section 3 for further details), the co-occurrence of other compounds targeting topoisomerases may reasonably change the overall topoisomerase-dependent genotoxic effects of *Alternaria* mycotoxins. Several studies demonstrated the ability of some polyphenols to poison topoisomerase I and/or topoisomerase II, albeit their specific mechanism of action has been poorly investigated. Kaempferol and quercetin were reported to be, at specific concentrations, non-covalently binders of topoisomerase IIα, while myricetin showed the ability to covalently bind to topoisomerase IIα and cleaving DNA in a redox-dependent way [132]. Additionally, the flavonoids quercetin, myricetin, fisetin, and apigenin were highlighted by other authors as poisons of topoisomerase I [133], whilst genistein, daidzein, biochanin A, chrysin, have shown poisoning effects also against topisomerases II [134]. Interestingly, genistein and especially delphinidin (that acts as catalytic topoisomerase inhibitor) were found to protect cells from AOH-induced genotoxicity [114]. Grapes and red wines are characterized by a large amount of resveratrol, belonging to polyphenols’ stilbenoids group, which has always been regarded to have beneficial effects thanks to its manifold activity. Nevertheless, the capability to establish non-covalent cross-linking interactions with both topoisomerase II and DNA leading to cell death was described too [135]. An influence of these compounds on poisoning and/or inhibition of topoisomerases by *Alternaria* mycotoxins, also diversifying the outcomes in vivo in a mixture-dependent way, appears therefore to be possible.

On the basis of the data reported above, *Alternaria* mycotoxins and a wealth of food constituents may interfere to each other, mutually influencing their final effects. Moving further steps toward a more precise molecular-oriented understanding of the food-specific and mixture-dependent outcomes in vivo will allow mapping those categories of food might pose a higher risk for specific toxicological endpoints. In the near future, adopting such an approach will effectively pave the ground to set personalized risk/benefit assessment studies of food prone to be contaminated by *Alternaria* mycotoxins.

## 5. Conclusions and Future Perspectives

*Alternaria* mycotoxins are frequently occurring in various fresh and processed foods such as cereals, fruits, vegetables, nuts, fruit and vegetable juices, seeds and oils. In many cases, contaminated foods have been found to simultaneously contain more than one *Alternaria* mycotoxin. In addition, the co-occurrence of *Alternaria* mycotoxins along with *Fusarium*, *Penicillium* and *Aspergillus* mycotoxins is also well documented, though not routinely checked. In addition, mycotoxins co-occur with the huge number of food constituents inherently present in contaminated foods. Notably, a growing number of data pointing to significant effects of chemical mixtures of mycotoxins in combination with each other or with food components is available. On this basis, a more precise description of mycotoxin contamination in food, detailing both the co-occurrence of mycotoxins and the types of co-contaminated food categories, is urgently required to better support risk assessment studies.

In this respect, the current risk assessment of mycotoxins is mostly based on human exposure data and animal toxicity evidences of individual compounds, while the evaluation of possible effects due to chemical mixtures is only occasionally assessed. Studies on the combinatory effects of different *Alternaria* mycotoxins, also in combination with other mycotoxins, have already shown that the co-exposure may result in either additive, synergistic, or antagonistic effects, depending on the doses, time of exposure or type of combinations assessed. In addition, recent findings have shown that mycotoxins may interplay with other food constituents, with different outcomes depending on the nature of combinations tested. Taken together, these results show that the toxicity of mycotoxins may significantly change depending on the composition of chemical mixtures, whereby not only co-contaminants but also food bioactives might act as contributors. This evidence pointed out the need to carefully check the multiple co-occurrence of mycotoxins, also in combination with the other food constituents. On the other side, it is crucial to characterize the effects the various combinations with the other food constituents may cause on the toxicity of mycotoxin mixtures. However, the evaluation of combinatory effects is not easy to perform since the toxic action exerted by individual mycotoxins is often strictly dependent on the cellular model and the concentrations tested. In addition, the use of different cellular models and different tested concentrations makes the inter-laboratory comparison of results difficult. Moreover, from a practical point of view, the number of food constituents possibly co-occurring with mycotoxins and potentially able to modulate their toxicity is so huge to make the systematic assessment of any possible combination unaffordable. Therefore, the definition of a consensus to define the combinations that really deserve investigations is strongly suggested. From a toxicological point of view, the use of the Adverse-Outcome-Pathway (AOP) approach or the adoption of grouping criteria, such as read-across methodologies or other computational-based categorizing methods, might provide a convincing rational to support the early definition of combinations to be tested. Moreover, in order to improve the interpretability of the data, homogeneity in the expression of the results, as well as in the tested concentrations, used cellular models, and applied methods, should become a common objective for researchers dealing with these issues in the future.

In summary, *Alternaria* toxins in food are not yet regulated mainly as a consequence of the shortage of toxicological occurrence and exposure data. A more in-depth elucidation of their toxicity, taking into account the effects of chemical mixtures, will ensure a more precise evaluation of their effects on human health eventually resulting in a more reliable assessment of risks with an overall lower degree of uncertainty. In this framework, this review collected the main data available so far in terms of occurrence and combined actions of *Alternaria* mycotoxins and it highlighted that chemical mixture may significantly change the individual toxicity of mycotoxins. Notably, most of the combinations found naturally in food still need to be tested in terms of toxicity. Therefore, it is hard to infer with precision the actual toxicological effects due to the consumption of food contaminated by *Alternaria* mycotoxins. Nonetheless, the data presented here may serve as a ground to design further studies to deepen the knowledge about the toxicity of this class of mycotoxins and to support the assessment of risk taking into account the actual role of chemical mixtures. The proposed paradigm can be logically extended to the risk assessment of other mycotoxins, as the relevance of mixtures has been described also for other classes of mycotoxins.

## Figures and Tables

**Table 1:** Co-occurrence of *Alternaria* toxins in food.

Food/Foodstuff	*Alternaria* Mycotoxins							Reference
	AOH	AME	ALT	TeA	TEN	ATX-I	Other	
**Fruits, Vegetables and Derivatives**								
Apple	X	X	X	X		X		[19]
Apple juice	X	X						[36,43]
Apple juice (concentrated)	X	X						[44]
Apple-pear-cherry (puree infant formula)	X	X		X	X	–	ALP (–)	[21]
Berry juice	X	X			–			[37]
Cherry-banana (puree infant formula)	X	X		X	–	–	ALP (–)	[21]
Cranberry juice	X	X						[39]
Cranberry nectar	X	X						[43]
Citrus juice	–	X ^a^		X ^a^	–			[38]
Grape juice	X	X						[39]
Ketchup	X ^a^	–	X ^a^					[45]
Ketchup	X	X		X	X			[38]
Mixed juice (fruits and vegetables)	X	X ^a^	X ^a^	X		–	Iso-ALT (–), AAL TA1 (–), AAL TA2(–)	[46]
Orange juice	X	X						[36]
Pepper	X	X		X				[47]
Prune nectar	X	X						[43]
Soya beans	X	X						[48]
Strawberry	X ^a^	X ^a^						[49]
Sweet pepper	X	X	X	X				[29]
Tangerine (flavedo)	X	X						[50]
Tomato	X	X	X	X		–		[19]
Tomato	X ^a^	X ^a^		X ^a^		–	ATX-II (–)	[51]
Tomato	X	X	X					[45]
Tomato (dried)	X ^a^	X ^a^	X ^a^		X ^a^			[52]
Tomato (puree and ketchup)	X	X	–	X	X ^a^	–	Iso-ALT (–), AAL TA1 (–), AAL TA2(–)	[46]
Tomato (sun-dried)	X	X	X					[45]
Tomato juice	–	X		X	X			[38]
Tomato sauce	X	X	X ^a^	X	X ^a^	X	ALP (X), AOH-3-S (X ^a^), AME-3-S (X)	[20]
Tomato sauce (puree infant formula)	X	X		X	X	–	ALP (–)	[21]
Tomato soup (puree infant formula)	–	X		X	–	–	ALP (–)	[21]
Vegetable juice	X	X						[36]
**Cereals and Derivatives**								
Bakery products (wheat- and rye- based)	X	X	–	X	X	–	Iso-ALT (–), AAL TA1 (–), AAL TA2(–)	[46]
Bread	X ^a^	X		X	X			[53]
Cereal grains	X ^a^	X ^a^	–		X ^a^			[41]
Corn silage	X ^a^	X ^a^			X ^a^		MACRO (X ^a^)	[33]
Dried noodles	X ^a^	X		X	X			[53]
Maize-based snacks	X	X						[54]
Millet (infant formula)	X	X		X	X	–	ALP (–)	[21]
Oat (infant formula)	–	X		X	X	–	ALP (–)	[21]
Ragi	–	X	X	X		–		[30]
Rice (infant formula)	X	X		X	X	–	ALP (–)	[21]
Sorghum	–	X	X	X		–		[30]
Spelt (infant formula)	X	X		X	X	X	ALP (–)	[21]
Wheat	X ^a^	X ^a^	X ^a^	X ^a^				[31]
Wheat	X ^a^	X ^a^		X ^a^				[55]
Wheat (infant formula)	X	X		X	X	X	ALP (–)	[21]
Wheat flour	X ^a^	X		X	X			[53]
Wheat flour	X ^a^	X	–	X	X ^a^	X	ALP (X), AOH-3-S (-), AME-3-S (-)	[20]
Wheat silage	X ^a^	X ^a^			X ^a^		MACRO (X ^a^)	[33]
Weathered wheat	X	X		X				[56]
**Dried Fruits and Nuts**								
Almonds	X ^a^	X ^a^			X ^a^			[57]
Almonds	X ^a^	X ^a^			X ^a^	–	MACRO (X ^a^)	[34]
Chestnuts	X ^a^	X ^a^			X ^a^			[57]
Dried figs	X ^a^	X ^a^			–			[57]
Dried grape berries	X	X	X	X	X	X	ATX-II (X), MACRO (X)	[35]
Dried jujubes	X ^a^	X ^a^			X ^a^			[57]
Dried persimmons	X ^a^	X ^a^			–			[57]
Dried raisins	X ^a^	X ^a^			–			[57]
Dried raisins	X	X	–	X	–			[58]
Dried wolfberries	X ^a^	X ^a^	–	X	X			[58]
Hazelnuts	X ^a^	X ^a^			X ^a^			[57]
Hazelnuts	X	X			X	X ^a^	MACRO (X)	[34]
Peanuts	X ^a^	X ^a^			X ^a^	–	MACRO (X ^a^)	[34]
Pine nuts	X ^a^	X ^a^			X ^a^			[57]
Pistachios	–	–			–	–	MACRO (X ^a^)	[34]
Walnuts	X ^a^	X ^a^			X ^a^			[57]
**Other Food and Foodstuff**								
Beer	X ^a^	–	X ^a^	–	X ^a^			[40]
Food supplement (antioxidants)	X ^a^	X ^a^		X ^a^	X ^a^			[42]
Food supplement (milk thistle)	X	X		X	X			[42]
Food supplement (phytoestrogens)	X	X		X ^a^	X			[42]
Red wines	X	X						[39]
Sesame seeds	X	X		X				[59]
Sunflower seed oil	X	X	–	X	X	X ^a^	ALP (X ^a^), AOH-3-S (-), AME-3-S (-)	[20]
Sunflower seeds	–	–	–	X ^a^	X ^a^			[41]
Sunflower seeds	X	X		X				[60]
Sunflower seeds	X	X	X ^a^	X	X	X ^a^	Iso-ALT (X ^a^), AAL TA1 (–), AAL TA2(–)	[46]
Sunflower seeds	X ^a^	X ^a^		X	X		ALTSOH (X ^a^), Val-TeA (X ^a^)	[61]
Vegetable oils (rapeseed and sunflower seeds)	X	X	–	X ^a^	X	–	Iso-ALT (–), AAL TA1 (–), AAL TA2(–)	[46]
White wines	X	X						[39]
Wines	X ^a^	–	–	X ^a^	–			[41]

X: certain co-occurrence; X ^a^: uncertain co-occurrence; –: checked but not detected.

**Table 2:** Co-occurrence of *Alternaria* toxins with other mycotoxins.

Food/Foodstuff	Co-Occurring Mycotoxins				Reference
	2 Mycotoxins	3 Mycotoxins	4 Mycotoxins	>4 Mycotoxins	
Berry juices	AFB2, AME AFB2, AOH AOH, AME	AFB2, AFG2, AOH	AFB2, AFG2, AME, AOH AFG1, AFG2, AME, AOH AFB2, AFG1, AFG2, AME		[37]
		AFB2, AOH, OTA			
		AFB2, AFG2, AME			
		AFB2, AFG2, AOH			
		AFG2, AME, AOH			
		AFB2, AME, AOH			
Sweet pepper (*Capsicum annuum*)	AFB1, TeA	ZEN, TEN, TeA	FB2, TEN, TeA, ZEN	*n* 16 (NIV, AOH, TeA, HT-2, FB2, OTA, T-2, FB1, TEN, AME, AFB1, DON, AFG1, AFB2, AFG2 and ZEN)	[29]
Durum wheat	n.a.	n.a.	n.a.	*n* 7 (EN B, EN B1, EN A1, AME, DON, HT2 and T2)	[67]
Dried fruits (raisins, dried apricots, dates and wolfberries)	TeA, MPA TeA, TEN	n.a.	n.a.	n.a.	[58]
Maize-based snacks	n.a.	n.a.	n.a.	*n* 6 (FB1, FB2, FB3, BEAU, AME, EMOD)	[54]
Nuts and dried fruits	AFB2, TEN	AFB2, TEN, AME	ZEN, TEN, AOH, AME	*n* 8 (AFB1, AFB2, ENB, ENB1, OTB, TEN, AOH, AME)	[57]
	AFG1, AME	AFB2, AOH, AME	ENA1, ENB, TEN, AME		
	ZEN, AOH	ENA1, ENB1, AME	AFB2, TEN, AOH, AME		
	BEA, AME	BEA, AOH, AME	AFB1, BEA, AOH, AME		
	T-2, AME	T-2, BEA, AME	AFG1, AFG2, ENB1, TEN		
	ENB1, TEN	AFB2, ENB, AOH	AFG1, ENB1, AOH, AME		
Beer	AOH, ZEN	n.a.	Ergometrine, AOH, ZEN, DON	n.a.	[64]
Food supplements (milk thistle - based)	n.a.	n.a.	AOH, AME, TEN, MPA Other	*n* 14 (AOH, AME, TEN, 3-ADON, FUS-X, ENN-B, ENN-B1, ENN-A, ENN-A1, BEA, DON, HT-2, T-2, ZEN) etc.	[42]

n.a.: information not available.

**Table 3:** LD_50_ values of *Alternaria* mycotoxins currently available.

Mycotoxin	Animal Species	Route of Exposure	LD_50_ (mg/kg b.w.)	Reference
AOH	Mouse (DBA/2)	intraperitoneal	>400 ^1^	[69]
AME	Mouse (DBA/2)	intraperitoneal	>400 ^1^	
TeA	Mouse	intravenous	115 (female)	[70]
			162 (male)	
		oral	81 (female)	
			186 (male)	
	Mouse (ICR)	intravenous	125 (male)	[71]
		intraperitoneal	150 (male)	
		subcutaneous	145 (male)	
		oral	225 (male)	
	Rat	intravenous	157 (female)	[70]
			146 (male)	
		oral	168 (female)	
			180 (male)	
	Chicken embryo	injection	548 ^2^	[72]
	White leghorn chicken	oral	37.5 ^3^	[73]

^1^ LD_50_ values of AOH and AME were not reached at the maximum dose tested, corresponding to 400 mg/kg, ^2^ Unit of measurement: µg/egg, ^3^ Information about sex not available.

## Supplementary Materials

The following are available online at https://www.mdpi.com/2072-6651/11/11/640/s1, Table S1: Co-occurrence of *Alternaria* toxins in food.
